# A community-based intervention for primary prevention of cardiovascular diseases in the slums of Nairobi: the SCALE UP study protocol for a prospective quasi-experimental community-based trial

**DOI:** 10.1186/1745-6215-14-409

**Published:** 2013-12-01

**Authors:** Samuel O Oti, Steven JM van de Vijver, Catherine Kyobutungi, Gabriela B Gomez, Charles Agyemang, Eric P Moll van Charante, Lizzy M Brewster, Marleen E Hendriks, Constance Schultsz, Remare Ettarh, Alex Ezeh, Joep Lange

**Affiliations:** 1 African Population and Health Research Center, PO Box 10787–00100, Nairobi, Kenya; 2 Department of Global Health, Academic Medical Center, University of Amsterdam and Amsterdam Institute for Global Health and Development, PO Box 22700, 1100 DE Amsterdam, The Netherlands; 3 Department of Public Health, Academic Medical Center, University of Amsterdam, Meibergdreef 9, Amsterdam 1105 AZ, The Netherlands; 4 Department of Family Medicine, Academic Medical Center, University of Amsterdam, Meibergdreef 9, Amsterdam 1105 AZ, The Netherlands; 5 Department of Internal and Vascular Medicine, Academic Medical Center, University of Amsterdam, Meibergdreef 9, Amsterdam 1105 AZ, The Netherlands

**Keywords:** Prevention, Cardiovascular risk factors, Cost-effectiveness, Slums, Sub-Saharan Africa

## Abstract

**Background:**

The burden of cardiovascular disease is rising in sub-Saharan Africa with hypertension being the main risk factor. However, context-specific evidence on effective interventions for primary prevention of cardiovascular diseases in resource-poor settings is limited. This study aims to evaluate the feasibility and cost-effectiveness of one such intervention—the “Sustainable model for cardiovascular health by adjusting lifestyle and treatment with economic perspective in settings of urban poverty”.

**Methods/Design:**

*Design*: A prospective quasi-experimental community-based intervention study.

*Setting*: Two slum settlements (Korogocho and Viwandani) in Nairobi, Kenya.

*Study population*: Adults aged 35 years and above in the two communities.

*Intervention*: The intervention community (Korogocho) will be exposed to an intervention package for primary prevention of cardiovascular disease that comprises awareness campaigns, household screening for cardiovascular diseases risk factors, and referral and treatment of people with high cardiovascular diseases risk at a primary health clinic. The control community (Viwandani) will continue accessing the usual standard of care for primary prevention of cardiovascular diseases in Kenya.

*Data*: Demographic and socioeconomic data; anthropometric and clinical measurements including blood pressure. Population-based data will be collected at the baseline and endline—12 months after implementing the intervention. These data will be collected from a random sample of 1,610 adults aged 35 years and above in the intervention and control sites at both baseline and endline. Additionally, operational (including cost) and clinic-based data will be collected on an ongoing basis.

*Main outcomes*: (1) A positive difference in the change in the proportion of the intervention versus control study populations that are at moderate or high risk of cardiovascular disease; (2) a difference in the change in mean systolic blood pressure in the intervention versus control study populations; (3) the net cost of the complete intervention package per disability-adjusted life year gained.

*Analysis*: Primary outcomes comparing pre- and post-, and operational data will be analyzed descriptively and “impact” of the intervention will be calculated using double-difference methods. We will also conduct a cost-effectiveness analysis of the intervention using World Health Organization guidelines.

**Discussion:**

The outcomes of the study will be disseminated to local policy makers and health planners.

**Trial registration:**

Current controlled trials ISRCTN84424579

## Background

The burden of cardiovascular diseases (CVD) is rising in sub-Saharan Africa (SSA) where up to 12.5% of deaths are attributable to CVD (SSA) [1,2]. Hypertension is the leading risk factor for CVD worldwide, and it is becoming even more pronounced in SSA [3]. For example, the average blood pressure of people in Kenya has risen from approximately 125 mmHg in 1990 to around 130 mmHg in 2010 [4]. This is in contrast to countries in North America and Western Europe where the weighted average blood pressure has decreased by approximately 3 mmHg in the same period [5]. This is partly because countries in SSA are mostly in an earlier phase of the epidemiological transition [6,7] in which relatively low levels of behavioral CVD risk factors are increasing rapidly compared to their counterparts elsewhere in the world [8,9]. On the other hand, countries in SSA also score relatively poorly in terms of availability of and access to medication for treating CVD and their risk factors [10].

Moreover, the health and economic impact of CVD in SSA and other low-resource settings is disproportionately higher than elsewhere [11]. Not only do people in SSA who suffer from CVD have a higher chance of disability or death, they are also more likely to have developed CVD earlier in life—during their most economically productive years [12,13]. At the same time, most countries in SSA are still struggling with a high burden of infectious diseases such as malaria and HIV/AIDS. The so-called risk of a “double burden of disease” due to infectious and non-communicable diseases poses a serious threat to the weak health systems in such resource-poor settings [14]. Hence, there is an urgent need to implement and evaluate cost-effective interventions for primary prevention of CVD in such settings. Generally, primary prevention of CVD could involve lifestyle interventions targeting the common behavioral risk factors for CVD—tobacco use, alcohol misuse, unhealthy diet and lack of adequate physical activity [15]. Other primary prevention strategies target the physiological risk factors for CVD including drug therapy for the treatment and control of high blood pressure, glucose and cholesterol [15]. While there is strong evidence of the benefits of lifestyle modification efforts in individuals at ‘high risk’ , the evidence of such interventions when implemented at a population level (including those at ‘low risk’) is less convincing [16].

Modeling studies have estimated that scaling up the coverage of appropriate drug therapy will be very cost-effective in reducing the burden of CVD in low-resource settings [17]. Specifically, it appears that improving availability of appropriate medication for people with hypertension may play a crucial role in slowing down the rising trends of CVD mortality in SSA [18]. However, in order to be successful, the scaling up of antihypertensive medication and indeed other cost-effective interventions for primary prevention of CVD needs to overcome certain barriers at both the population and individual levels. First, the level of awareness about hypertension and other CVD risk factors is low, and in most countries in SSA screening opportunities at the population level are limited [7,19,20]. At the individual level, access to quality treatment and follow-up care for hypertension and CVD in general remains poor [21,22]. Primary health care facilities in SSA often lack essential medicines and technologies for diagnoses and treatment of hypertension and other CVD risk factors [23]. In addition, standard treatment guidelines or protocols for CVD are usually lacking and/or not implemented [24].

Therefore, health systems in most countries in SSA might not be prepared to deal with a hypertension or CVD epidemic even though cost-effective interventions are available. This is more so among the urban poor who are resident in vast slums across the sub-continent. There is a paucity of information on CVD and its risk factors in slums in SSA. Yet, more than 60 percent of urban populations in the sub-continent reside in slums or informal settlements [25]. For instance, in Nairobi (Kenya), up to 70 percent of the urban population resides in vast slums across the city [26]. These slums are typically underserved by social amenities including access to quality health care. Some evidence suggests that such populations fare worse than their non-slum and even rural counterparts on most health measures including high prevalence of CVD and low levels of awareness, treatment and control of hypertension [27].

The “Sustainable model for Cardio-vascular health by Adjusting Lifestyle and treatment with Economic perspective in settings of Urban Poverty” (acronym: SCALE UP) study is designed to implement a comprehensive intervention package of primary prevention strategies for CVD risk reduction in a slum setting in Nairobi, Kenya, and to evaluate its feasibility and cost-effectiveness. Specifically, the intervention package integrates approaches that aim to raise awareness and improve detection of hypertension at the population level. Additionally, the intervention aims to provide access to standardized quality treatment and follow-up for hypertensive patients with the overall objective of reducing their CVD risk profile in such a low-resource setting.

## Methods/Design

### Design

SCALE UP is a community-based intervention aimed at reducing cardiovascular risk in people free from cardiovascular disease. It is designed to allow for a before-after comparison of cardiovascular risk between a control and an intervention setting.

### Setting

Since 2002, the African Population and Health Research Center (APHRC) has been operating the Nairobi Urban Health Demographic Surveillance System (NUHDSS). Details about the NUHDSS have been provided elsewhere [28]. In brief, the Demographic Surveillance Area (DSA) covers two socio-demographically similar slums (Korogocho and Viwandani), each located about 5 to 10 km from Nairobi (Kenya). There are approximately 72,000 individuals resident in 25,000 households almost equally distributed in both slums. High levels of poverty, unemployment and lack of social amenities, including limited access to quality primary health care, characterize both slums. Specifically, there are only two public primary health facilities located on the outskirts of either slum. However, there are numerous private health providers in the slums, the majority of which are unlicensed and unregulated. Most of the private facilities operate largely for profit and rarely provide professional quality care. However, slum residents seem to prefer these services to the public ones for a number of reasons, including easier access, more approachable staff and flexible working hours (APHRC unpublished observations).

### Intervention community

Korogocho slum, which has eight villages, each with between 3,000–5,000 residents, will be the intervention site. This slum has one centrally located private health facility with a reliable track record of providing primary health services. This facility is known as *Provide International Clinic*. It is well known in the slum and, although most patients still make out-of-pocket payments for services received, the clinic offers non-profit services at highly subsidized (through donor funding) costs to residents. There are no physicians or medical officers present at this clinic. Nurses and clinical officers (health personnel with a diploma in clinical medicine) provide consultations. However, the facility does not typically provide primary preventive services for CVD such as screening and treatment of hypertension. The location of this clinic and the absence of primary preventive services for CVD guided our choice of intervention site.

### Control community

Viwandani slum will be the control site. There are seven villages in Viwandani with approximately 2,000–4,000 residents each. Unlike Korogocho, Viwandani does not have a centrally located health facility. The main health facility serving Viwandani is located on the outskirts of the slum. This facility is publicly owned and represents the usual standard of care for CVD that is available to underserved slum communities in Nairobi, thus making Viwandani the appropriate ‘control’ site in comparison with Korogocho. The clinic in Viwandani operates a weekly CVD clinic where approximately 30 patients with uncomplicated hypertension and/or diabetes from Viwandani slums are seen on each clinic day. Like the facility in Korogocho, the Viwandani clinic is also run by nurses and clinical officers.

### Study population

#### Inclusion criteria

Adults aged 35 years and above living in the slums of Korogocho and Viwandani who give informed consent to participate in the study. The main reason for this age cutoff is because the group above 35 years represents 21% of the total population and accounts for 71% of all known hypertensive cases, according to a CVD risk factor survey conducted in the DSA in 2008 (APHRC unpublished data). Also, due to financial constraints, the study could not be extended to an unrestricted age group. Persons with diagnosed hypertension and/or on antihypertensive therapy will be included in the study.

#### Exclusion criteria

The following will be excluded from the study: pregnant women, persons with self-reported pre-existing CVD (myocardial infarction, stroke, heart failure and angina) and all those unable to provide informed consent such as the mentally incapacitated.

### The intervention package

**Figure 1:**
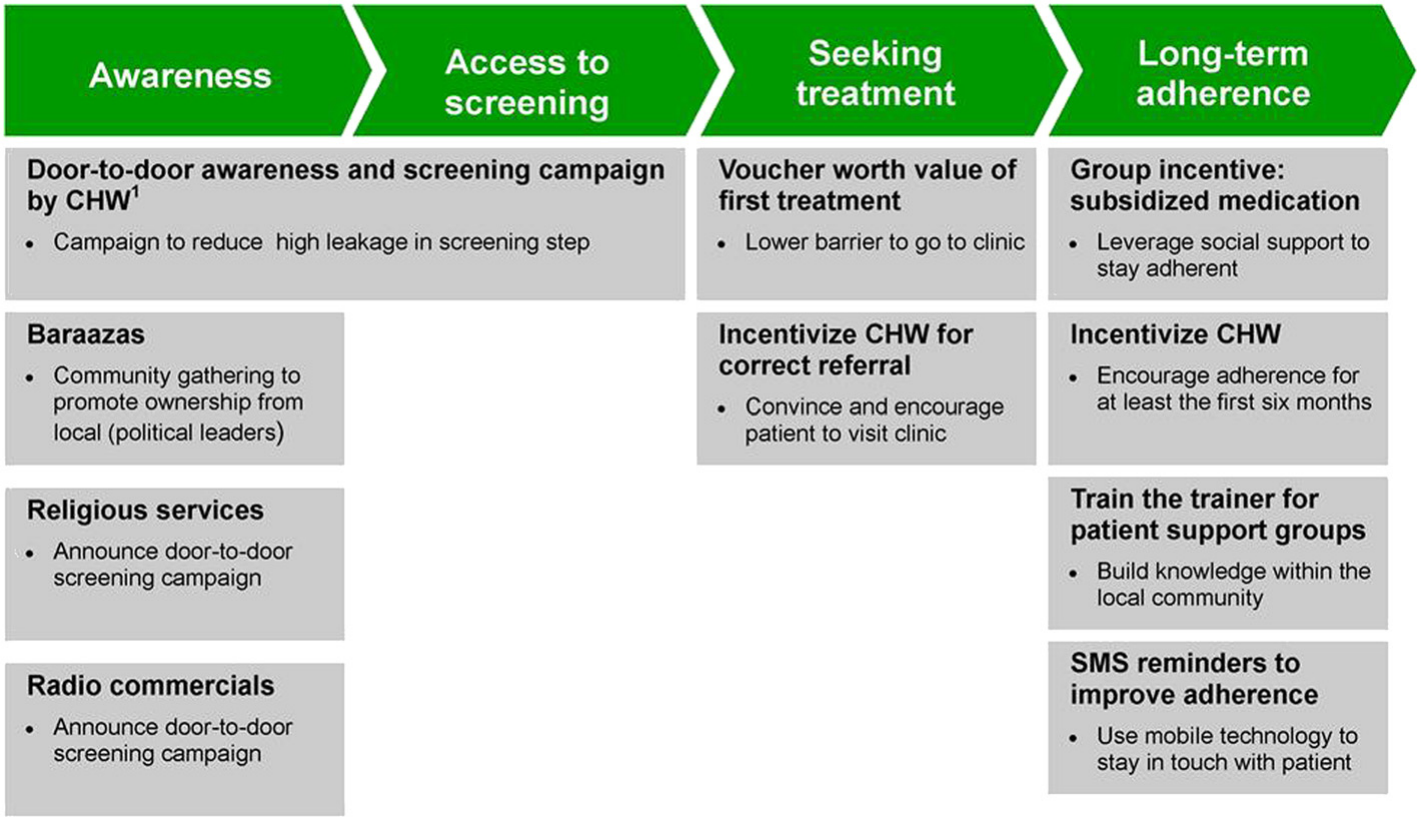
**Overview of interventions in the SCALE UP intervention package.**^*1*^*CHW*: *Community health worker*.

APHRC and the Amsterdam Institute for Global Health and Development (AIGHD) have developed an intervention package for primary prevention of CVD in urban slums based on findings from earlier studies by APHRC on CVD risk factors in this specific setting [29], literature review [30] and input from various experts and local stakeholders. The intervention package is composed of four components, which will be described below (see Figure 1).

#### (1) Raising awareness and (2) improving access to screening

The first two components of the intervention package include door-to-door household visits by community health workers (CHWs) to raise awareness about CVD risk and to conduct screening of each eligible adult aged 35 years and above in the intervention slum in order to determine their individual CVD risk profile. CHWs are typically community-based volunteers who have an interest in community health. They are usually well known by the community and most have received some form of basic training in community health activities such as peer health education for HIV/AIDS prevention. The SCALE UP study will recruit CHWs to represent each of the eight villages in the intervention slum. These CHWs will then be trained and equipped to perform the door-to-door visits and screening exercise in their respective villages in the intervention slum.

Two weeks prior to the commencement of the screening exercise, there will be public awareness campaigns organized by the CHWs in each village of the intervention slum. This awareness campaigns will take place at community gatherings (known locally as *barazaas*). At these *barazaas*, CHWs together with village leaders will inform the audience about the burden of CVD in the community and the need to participate in the door-to-door screening. Additionally, CHWs will visit religious gatherings, usually held at local churches and mosques, to provide the same information. Such gatherings are usually well attended by respected members of the community who will then pass the information on to others. Finally, radio jingles announcing the door-to-door screening will be aired daily on the local radio station (Korogocho FM) over a 2-week period from the start of the intervention.

The SCALE UP study investigators will train the CHWs to assess the study participants’ level of engagement in risky lifestyle behavior including tobacco use, alcohol misuse, physical activity levels and dietary habits. CHWs will also be trained in performing basic anthropometric and clinical measurements including height, weight, waist and hip circumference, blood pressure and blood glucose. They will be provided with the appropriate equipment for the clinical measurements (see Table 1). Also, each CHW will be trained to provide brief counselling assistance (BCA) of healthy lifestyle modification using the six As approach –*Ask*, *Advice*, *Assist*, *Arrange*, *Agree and Affirm*[31]. Traditional BCA does not include the sixth A (Affirm) as a separate entity. However, the SCALE UP team has included this component to emphasize the need for CHWs to encourage study participants to continue with any healthy lifestyle behavior in which they reported being currently engaged. All eligible and consenting study participants will receive BCA during the door-to-door visit. If the eligible adult(s) are not at home, the CHWs will try to visit another time, with a maximum of two attempts.

**Table 1:** List of screening equipment

**Equipment**	**Units**
SECA 201 circumference measurement tape	*cm*
SECA 874 flat scale electronic	*kg*
SECA 214 stadiometer transportable	*cm*
OMRON M6 blood pressure machine	*mmHg*
ACCUCHECK glucometer and test strips	*mmol*/*l*

#### (3) Facilitating access to quality treatment

The third component of the intervention package includes facilitating access to quality treatment for hypertension. During the door-to-door screening all persons with elevated blood pressure (≥140 mmHg systolic and/or ≥90 mmHg diastolic) will be referred to the intervention clinic—Provide International Clinic—by the CHWs. A project supervisor will independently visit each person referred by a CHW to perform a confirmatory blood pressure check before that person proceeds to the intervention clinic. Those people below 35 years who are interested in knowing about their blood pressure and/or CVD risk will be referred to a central screening point in the slum. The results of this additional screening will not be considered as part of this study.

Based on previous experience in the slums, the SCALE UP team included two incentives in the intervention package to encourage referred study participants to seek care at the intervention clinic. First, each referred participant will receive a voucher for a first free consultation at the clinic. At the same time, CHWs will receive a cash reward of approximately 3 US dollars (US$ 3.00) for each of their referred participants who attends the clinic for the first time. Hopefully, this cash reward will be a reasonable incentive for each CHW to follow up those whom they have referred and encourage them personally to attend the clinic for the first time.

In addition to improving access to treatment, it is also important to ensure that the treatment is of high quality. Thus, as part of the intervention package, the SCALE UP team will build the capacity of the intervention clinic to provide primary care services for CVD risk management. To this end, the intervention clinic will be equipped with basic and essential diagnostic equipment for CVD including a validated digital blood pressure measurement device, measurement tape, weighing scale, height measurement board, glucometer and blood glucose test strips. These devices have been selected in accordance with WHO essential technologies and tools for implementing non-communicable disease (NCD) interventions in primary health care using non-physician health workers [32].

Selected non-physician health workers including clinical officers and nurses from the intervention clinic will be trained on how to implement a standard protocol for management of hypertension at the primary health care level in low-resource settings. This protocol was developed by the SCALE UP study team in collaboration with senior cardiologists with experience in primary care from the University of Nairobi and the Division of Non-Communicable Diseases in the Kenya Ministry of Health (see Additional file 1). The various treatment thresholds for hypertension given in the protocol are based on a non-laboratory-based CVD risk prediction assessment method [33]. Using this method, all participants with high blood pressure who enroll at the intervention clinic will be classified as low (≤10%), moderate (>10-20%) or high (>20%) risk based on their individual 10-year CVD risk (fatal and non-fatal) profile. Treatment for hypertension will range from lifestyle modification to use of locally available antihypertensive medication depending on the risk profile of the participants. The treatment threshold and target blood pressure are specified in Additional file 1.

In addition to referring participants with high blood pressure to the intervention clinic, CHWs will also be instructed to refer other potentially moderate to high CVD risk study participants. Specifically, CHWS will be instructed to perform random blood glucose tests on all study participants who are older than 55 years of age AND have any of the following: systolic blood pressure ≥140 mmHg, diastolic blood pressure ≥90 mmHg OR abnormal waist circumference (>102 cm for men and >88 cm for women). Participants whose random blood glucose is ≥11.1 mmol/l will be referred to the intervention clinic for follow-up. At the clinic, random blood glucose will be repeated, and if still ≥11.1 mmol/l, the participant will be managed in accordance with the Kenya national clinical guidelines for management of diabetes mellitus [34]. As diabetes management is not a primary objective of the SCALE UP study, the intervention clinic will only be stocked with metformin—a relatively affordable oral hypoglycemic medication. Participants requiring other antidiabetic medication including insulin, based on the national guidelines, will be referred to the nearest district hospital.

#### (4) Promoting long-term adherence

The final component of the intervention package aims to promote long-term adherence among participants enrolled in the intervention clinic. For logistic reasons, all participants at the intervention clinic will be required to visit the clinic at least once every month for the entire duration of the study. Based on previous experience in the study area, longer follow-up periods tend to increase the chances of patient loss to follow-up. Beyond the regular follow-up interval, the adherence component of the intervention package will also include two subcomponents:

First, there will be an incentive-driven support group system for all participants enrolled into the intervention clinic. Each support group will have about 10–30 members drawn from participants living within the same villages. CHWs from respective villages will coordinate the activities of the support groups. This subcomponent of the intervention package seeks to leverage on the group dynamics of these support groups to promote adherence among participants. To this end, there will be a group incentive that rewards the entire group for achieving a collective level of adherence to clinic appointments at a level of 80% or more for a consecutive period of 6 months. If achieved, the entire group will receive a rebate in the monthly cost of their medication equivalent to approximately one-third of the usual cost. The estimated usual cost of medication for hypertension in the intervention clinic is Ksh 150 (US$ 1.8) per month. Additionally, the CHW will also receive a cash incentive to follow up every individual participant in the support group and encourage him/her to adhere to the clinic appointments. If participants remain adherent over the first 6 months of clinic enrollment, the CHW will receive a cash bonus of approximately US$ 1.8 per participant. Such an incentive is crucial because a previous study in the study areas found that almost 70% of hypertensive patients who drop out of primary health care clinics do so within the first 6 months (APHRC unpublished observations). The use of incentives to improve patient adherence has been tested in other settings, though the evidence is mixed [35].

There are other expected benefits that participants will enjoy for being part of the support group. The support group will hopefully be a forum where participants can share in the experiences of living with hypertension and learn from each other on how best to cope with this condition. Also, highly motivated participants will be selected by CHWs to become peer-educators. There will be train-the-trainer sessions where these participants will be trained by local experts on how to adopt healthy lifestyle changes such as healthy cooking classes and physical activity sessions, to mention a few.

The second subcomponent of the intervention package aimed at promoting long-term adherence is the use of the mobile phone Short Message Service (SMS). Unpublished data from the study area show that more than 80% of the adult population reports owning a mobile phone, and the remainder report having a close neighbor or other family member who owns a mobile phone through which they can be reached. Studies from other chronic conditions such as HIV have shown encouraging levels of success in the use of SMS to improve adherence [36]. In the SCALE UP study, an SMS will be sent every week to remind participants about their next clinic appointments, to take their medication and to provide them with healthy lifestyle tips.

### Data management

To measure the health effect of the whole intervention package at the population level, there will be two cross-sectional surveys (before and after, 12 months apart). Data will be collected on demographic and socioeconomic variables, behavioral risk factors such as tobacco and alcohol use, anthropometric measurements such as height and weight, and clinical measurements such as blood pressure and random blood glucose. Four cadres of field staff will be involved in data collection. These include field interviewers, CHWs, field assistants and supervisors.

Field interviewers will be trained by the SCALE UP study investigators to collect the demographic, socioeconomic and behavioral risk factor data using structured interviews during the cross-sectional surveys in both the intervention and control sites. In the intervention site, the anthropometric and clinical measurement will be performed by CHWs. In the control site, however, measurements will be performed by trained field assistants rather than CHWs. This is because CHWs are considered to be part of the intervention. Each interview is estimated to last approximately 30 min, followed by the physical and clinical measurements. The field staff will be instructed to follow standard procedures for all measurements as outlined in the WHO STEPS manual [37]. Specifically, while taking the blood pressure, field staff will be required to ensure that the respondent remains seated for about 5 min, with no talking, holding the monitoring device on the upper arm and holding it at heart level against his/her chest. The blood pressure will be measured three times, using the left arm. To minimize observer bias, validated digital equipment will be used, the OMRON M6® (Digital Automatic Blood Pressure Monitor). Note that referral of participants for further management will be based on the average of the three blood pressure measurements (systolic ≥140 and/or diastolic ≥90 mmHg).

At the intervention clinic, additional information will be collected on clinic attendees to monitor their progress and evaluate the effect of the intervention on blood pressure control and overall CVD risk profile. Baseline interviews will be conducted with each clinic attendee by trained field interviewers. Immediately after an interview, nurses at the clinic will record physical and clinical measurements for each clinic attendee. These measurements will be repeated each time the participant attends the intervention clinic over a period of at least 12 months. At the end of this period, an endline interview will be conducted with each clinic attendee. Table 2 summarizes the data to be collected over the intervention period.

**Table 2:** Data collection schedule

**Data**	**Baseline**	**Ongoing**	**Endline**
***Population based***	X		X
Socio-demographic characteristics	X		
CVD behavioral risk factors	X		X
Physical measurements (weight, height, waist and hip circumference, blood pressure)	X		X
Blood testing (glucose)	X		X
***Clinic based***			
Physical measurements (weight, height, waist and hip circumference, blood pressure)	X	X	X
Lifestyle modification advice		X	
Drug prescriptions and side effects		X	
Morisky adherence score		X	
***Operational data***			
Costs		X	
Timesheets		X	

All data will be entered onto electronic data collection forms pre-loaded into Mecer® Netbooks and stored in an SQL database managed by the data unit of APHRC. To ensure data quality, the field staff will be trained in interviewing techniques and how to use the Netbooks. Data collection forms will be translated into Swahili and back to English for consistency. Interviews will be conducted in Swahili—the main lingua franca in the study areas. Supervisors will be recruited to conduct spot checks of at least 5% of randomly selected interviews conducted by each field staff they supervise. They will also perform a random check of 5% of data collection forms completed by field staff. Forms will be checked for errors, missing information and inconsistent responses, and, where necessary, field staff will be required to revisit a study participant in order to clarify any erroneous information.

At the intervention clinic, qualified data entry clerks will be trained to double-capture all clinic data collected by the nurses and clinical officers during the patient consult. Clinic data will also be electronically captured using Netbooks.

Finally, cost data will be collected on an on-going basis in order to feed into the cost-effectiveness analysis of the intervention package. This will be done through an adapted checklist to collect information on costs and related time spent in all aspects of implementing the intervention package.

#### Primary outcomes

The primary outcomes of the SCALE UP study are:

1. The difference in change in the proportion of the study populations (intervention and control slums) that are at high risk of CVD (defined as >10% risk of developing cardiovascular event in the next 5 years based on the method for assessment of cardiovascular disease risk by Gaziano et al. [33]).

2. The difference in change in mean systolic blood pressure in the study populations (intervention and control slums).

3. The change in mean systolic blood pressure among participants attending the local clinic (intervention slum only).

4. The net cost of the intervention package per disability-adjusted life year gained (intervention slum only).

#### Secondary outcomes

1. Prevalence of hypertension in the intervention and control slums.

2. Proportion of hypertensive respondents who are on treatment, and under control, in the intervention and control slums.

3. Proportion of high-risk participants who sought first time treatment after screening and referral.

4. Prevalence of behavioral and biological CVD risk factors: smoking, physical exercise, diet, alcohol intake, body mass index, waist circumference and waist-to-hip ratio in the intervention and control slums.

### Sample size considerations

In order to detect a 5% reduction at endline in the proportion of adults aged 35 years and above who are at moderate or high risk of CVD [38,39] in the intervention population versus no change in the control population (assuming both populations have similar start prevalence at 25%), we need 2,927 respondents in both intervention and control sites, using an alpha of 0.05 and power (1-beta) of 0.90. Taking into account a non-response rate of 10%, we will approach 3,220 individuals per cross-sectional study—that is, 1,610 per site at baseline and endline surveys, respectively.

The sampling frame will be based on the most recently updated NUHDSS database. This database contains details of about 72,000 individuals including names, locations, gender, dates of birth and residential status in both slums. In the control site, we will use computer randomization (STATA® statistical software) to select the 1,610 individuals aged 35 years and older per site for each cross-sectional survey.

In the intervention site, the same computer randomization process will be followed. However, unlike the control site, the 1,610 individuals to be included in the cross-sectional survey analysis will be collected retrospectively. In other words, the intervention package will be delivered to all adults aged 35 years or older in the intervention site—that is, 6,780 individuals according to the DSS database (as at 15 June 2012).

At the clinic level, we calculated that in order to detect a 10 mmHg reduction in blood pressure (at 20 mmHg standard deviation, alpha of 0.05 and 1-β on 0.9), about 44 participants are needed. However, it is projected that approximately 1,350 participants (out of 6780) will be referred from the door-to-door visit. This number is derived from a 20% prevalence of hypertension among adults aged 35 years and older in the study area [27]. We estimate that roughly half of these 1,350 participants, being 675, will continue visiting the clinic for treatment. Hence, this number of people is more than sufficient for the analysis of our main primary outcome at the clinic level.

### Analysis

Due to the fact that the slums are part of a large demographic surveillance site and therefore not randomized, we will use the double-difference method [40] to evaluate the primary outcomes in the intervention versus control sites. In this approach, Impact = (Y_p(t>0)_ – Y_p(t=0)_) – (Yc_(t>0)_ – Yc_(t=0)_) where Y_p_ is the primary outcome in the intervention group and Y_c_ is the outcome in the control group. For the double-difference method to work, it is essential that there are at least two pre-intervention data points. In addition, having many preintervention data points allows for the detection of shifts or interruptions in trends (if any) after the introduction of an intervention. A cross-sectional study conducted in the NUHDSS from 2008–2009 provides one time point of pre-intervention data on CVD risk in the intervention and control slums [27]. Note that cardiovascular risk reduction will be calculated by entering the outcomes in a non-laboratory-based CVD risk assessment chart [33]. Regression analyses will also be performed to investigate the association of each risk factor with the main outcomes such as blood pressure. Specifically, multivariate analyses will be used to adjust for known or perceived confounding variables while comparing outcomes between intervention and control sites. Prior to this, descriptive statistics will be applied to compare characteristics of the intervention and control sites. For behavioral risk factor analysis, we will use an interpretive descriptive approach with matrix comparisons between groups (such as sex, age group and site).

Cost-effectiveness analysis will be conducted according to the WHO framework for cost-effectiveness analysis [41]. This framework will consider intervention effectiveness data based on changes in blood pressure and overall predicted cardiovascular risk at the population level, as well as cost data on the intervention. The overall cost-effectiveness of the intervention package will be calculated in terms of DALYs averted per US dollar.

Intervention cost will be estimated using a micro-costing approach where feasible. Micro-costing is a process of systematically identifying and measuring resource utilization using a process tracking system and interviews with the local program team [42]. According to the Panel on Cost-Effectiveness in Health and Medicine, the theory and process of valuing costs through a micro-costing methodology rest on a three-step approach: identification, measurement and valuation of resources used [43]. For other non-specific costs, gross costing methods will be considered. Once resource utilization has been measured, the component-specific costs of the intervention can be computed by multiplying the quantity of each type of resource consumed by unit costs. The component-specific costs can be summed up to get the total costs of the intervention [42]. Finally, the outcome of this analysis will be the average cost for CVD risk reduction per participant per year. Data on costs and timings will be collected from the preparation of the intervention until the end of the intervention period. A yearly discount rate of 3% will be used for long-term modeling and projections [44]. Estimations will be extended to project the cost-effectiveness of the intervention package were it implemented on a national scale.

Finally, in order to determine the scalability and feasibility of the intervention package, a comprehensive process evaluation will be conducted involving analysis of operational data as well as qualitative sub-studies with beneficiaries and other relevant stakeholders such as local policy makers.

Additionally, since the package is to be implemented in a private health sector setting, it will be important to examine essential aspects of the intervention package that are needed to translate the package to the public sector.

### Ethical approval

Ethical approval for this study was obtained from the Kenyan Medical Research Institute (KEMRI), reference KEMRI/RES/7/3/1 no. Non-SSC 399, dated 11 June 2012, renewable annually. Informed consent will be applied to all participants, and the overall study complies with the Declaration of Helsinki principles.

## Discussion

Although it is evident that a CVD epidemic is on the rise in most countries in SSA [10], there is limited evidence on the feasibility, cost-effectiveness and scalability of comprehensive primary prevention programs for CVD in these settings, in particular in the slum population. There is a scarcity of studies in SSA on community-based intervention for reduction of cardiovascular risk [30], and very few of them have looked into the cost-effectiveness [45] or scalability [46]. Considering the possibility of a double burden of disease that many countries in SSA are facing, it is essential to provide insight into the health impact and feasibility of primary prevention programs in order to enable policymakers and other stakeholders to make effective choices within their limited resources.

Despite the facts that the rise of CVD in SSA has been strongly linked with urbanization, and a sizable majority of the urban population in SSA is resident in slums, there are no specific studies or programs designed for this extremely vulnerable population. It is important to share knowledge and experiences on how this growing population at risk can be supported to reduce their cardiovascular burden. Therefore, it is important to implement programs in these challenging living circumstances and evaluate their feasibility and health effect. The SCALE UP study is unique in this regard.

In our intervention package we focus specifically on screening and treatment of hypertension as this is the main modifiable risk factor to achieve CVD risk reduction in SSA [47]. It is our hope that the combination of raised awareness through access to screening, improved access to quality treatment and the promotion of adherence will reduce hypertension rates in the study area to the extent that it is significantly detectable at population level. However, due to the depth of data that we will collect as part of the study, we will also be able to assess the effect of our intervention on behavioral risk factors for CVD such as tobacco use, excessive alcohol intake, poor dietary behavior and physical inactivity, as well as biological CVD risk proxies such as body composition (BMI) and blood glucose levels.

As mentioned previously, the content of our intervention arose from a theoretical cost-effectiveness analysis. Based on earlier studies done by APHRC and literature review. we made estimations of the different interventions possible in raising awareness, screening, treatment and promoting adherence in a slum setting. The final package is based on the theoretical effectiveness of each individual intervention component. Should our analysis prove the SCALE UP intervention be cost-effective, we will then work with local stakeholders and policymakers toward integrating the package in the larger health sector. Additionally, this vision of the scalability of the project not only implies the roll out of primary preventive services for CVD to more slums, but also provides the opportunity for the integration of other essential preventive services such as HIV testing or childhood vaccinations. Therefore, we anticipate and hope that this program will stimulate strengthening of weak health systems and structures in the ever-expanding slum settlements in SSA and beyond.

Our study is limited in the generalizability of the setting where the intervention package is tested. The DSA has been under surveillance for the past 10 years and may not be typical of other slum settings where the NUHDSS infrastructure does not exist. Another limitation of our study is that the intervention package cannot be evaluated in terms of its individual components. In other words, it will be impossible to tease out which parts of the intervention were most effective relative to others. However, we are confident that the logical composition of the complete intervention package will make it practical to implement as a whole in other similar settings should the multi-component intervention have an overall beneficial effect.

In conclusion, it is our hope that the outcomes of this study will inform policy makers and health professionals at various levels about the feasibility and cost-effectiveness of implementing community-based cardiovascular risk prevention programs in low- and middle-income countries, and specifically for the urban poor.

## Trial status

Participant inclusion started in August 2012 and participant recruitment and follow-up at the intervention clinic will continue until December 2013.

## Abbreviations

AIDS: Acquired immune deficiency syndrome; APHRC: African Population Health Research Center; AIGHD: Amsterdam Institute for Global Health and Development; BP: Blood pressure; CHW: Community health worker; CVD: Cardiovascular diseases; DALY: Disability adjusted life year; DSA: Demographic surveillance area; LMIC: Low- and middle-income country; NCD: Non-communicable diseases; NUHDSS: Nairobi Urban Health Demographic Surveillance System; RBS: Random blood sugar; SMS: Short message service; SSA: Sub-Saharan Africa; WHO: World Health Organization.

## Competing interests

The authors declare that they have no competing interests.

## Authors’ contributions

SO and SV both conceptualized the study. SO, SV, GBG, CK, JL, LB, MH, CS, EM, RE, CA and AE all participated in the study design and contributed to the writing of the study protocol, drafting and editing of this manuscript. JL is the overall study principal investigator, and CK is the co-principal investigator. All authors read and approved the final manuscript.

## Authors’ information

SO and SV are Senior Research Officers at APHRC, work as project managers of the SCALE UP project and are involved in a joint PhD program at the University of Amsterdam. CK and JL are part of the steering group of the SCALE UP study. GBG, CA, LB and EM give support from the AIGHD and University of Amsterdam. RE supports the project as a senior researcher at APHRC.

First authorship is shared by Samuel Oti and Steven van de Vijver.

## Supplementary Material

Additional file 1 SCALE UP guidelines for management of hypertension in primary care. Click here for file
