# Supplementary material for: A community-based intervention for primary prevention of cardiovascular diseases in the slums of Nairobi: the SCALE UP study protocol for a prospective quasi-experimental community-based trial
Source: Trials. 2013 Dec 1;14:409. doi: 10.1186/1745-6215-14-409 (PMC4220814; doi:10.1186/1745-6215-14-409)
Supplement: Additional file 1 — SCALE UP guidelines for management of hypertension in primary care. [file 1745-6215-14-409-S1.docx]

**SCALE UP GUIDELINES**

**FOR**

**MANAGEMENT OF HYPERTENSION IN PRIMARY CARE**

Preamble

These guidelines were developed in partnership with the University of Nairobi and the African Population and Health Research Center. The guidelines are based on international standards (mainly developed by the World Health Organization) that have been adapted to suit the context of primary health care in Kenya.

Settings

These guidelines are designed for low-resource primary health care settings and can be used by medical officers and other health workers (Qualified Nurse/Clinical Officer).

Minimum resource requirements

- Human resources: Qualified Nurse/Clinical Officer

- Equipment: Accurate validated blood pressure measurement device, measuring tape and weighing scale, equipment for testing blood glucose, urine sticks

- Drugs: Thiazide diuretic (e.g. hydrochlorothiazide), long acting Calcium Channel Blockers (e.g. nifedipine) Angiotensine-Converting Enzyme inhibitors (e.g. enalapril)

- Other facilities: System for maintaining medical records, referral facilities

Definitions

- Hypertension: elevated blood pressure sustained at a level equal to or above 140 mm Hg systolic or 90 mm Hg diastolic.

Classification

- Essential hypertension: not due to any known underlying cause, but risk factors include a family history for high blood pressure, stress, poverty, excessive alcohol intake, increased salt intake, a sedentary life style, and obesity.

- Secondary hypertension: can be caused by diseases affecting the renal or endocrine system, or by certain prescription medication such as steroids, hormonal contraceptives, non-steroidal anti-inflammatory drugs (NSAIDs).

About 95% of cases are classified as essential hypertension and are generally symptomless. However, secondary hypertension should be suspected in young patients (less than 30 years of age), patients whose blood pressure is not responding to medication, and those suffering from severe symptoms such as sweating, oedema, flushing, headache, and patients with protein or blood in urine.

Clinical significance

The primary reason for managing hypertension is not solely about regulating blood pressure but mainly to prevent cardiovascular diseases and other complications. Poorly managed hypertension can lead to heart attack, heart failure, strokes, renal problems, eye problems, and sexual dysfunction.

Screening

- Blood pressure: must be measured at every contact of an adult with a health worker. This applies specifically to every adult that has never had their BP measured and all persons older than 35 years that have not had their blood pressure checked in the last 12 months and/or have typical physical complaints (such as mentioned above).

- Blood glucose: among hypertensive patients, >55 years, waist circumference >88cm for women and >102cm for men, and/or typical physical complaints. If fasting blood glucose measured at finger prick is >6,1 mmol/L or random blood glucose >11,1 mmol/L act upon National Diabetes Guidelines.

Basic clinical evaluation

- History including age, gender, tobacco, physical activity, medication, alcohol, diet (salt intake, fruits and vegetables) and complaints suggestive of cardiovascular, cerebrovascular, ocular, and renal disease like pain on the chest, fatigue, paralysis, blurred vision, headaches, urinary problems

- Basic physical examination like heart sounds, arterial pulses, peripheral oedema, and pallor

Measurement techniques

- Blood pressure (in mmHg): an appropriate cuff size should be used with the position of the cuff at the level of the heart. When using digital machines they should be validated and frequently recalibrated. Patient should be sitting in upright position after 5 minutes of rest, with an empty bladder (feasible). Patient should not speak during the measurement. If BP>140/90, take a second measurement. If the second measurement is substantially different from the first (>5mmHg difference) for either systolic or diastolic blood pressure, take a third. Calculate the average between the two latest outcomes. This procedure should be observed in each subsequent clinic visit. First time: take BP on both arms and use arm with the highest reading for monitoring of BP. If difference is more than 20mm Hg, then refer.

If the average of the two latest measurements gives a BP>140/90 do another measurement in one week’s time.

- Waist circumference (in cm): midway between the lower rib margin and the upper side of the hip.

- Body Mass Index (BMI) = weight (kg)/height (m) ^2^.

Management

Lifestyle adjustment is an essential element of treatment as it can lower the blood pressure and also reduces overall risk of cardiovascular diseases. Proper control of lifestyle may even reduce or eliminate the need for drugs.

*Lifestyle adjustments*

To all patients:

- Stop smoking

- Diet (reduce total and saturated fat, reduce sugar, encourage fruits and vegetables)

- Reduction in salt intake (no more than one teaspoon or 5g salt/day)

- Physical activity (daily >30 minutes of moderate activity like brisk walking)

- Weight control (reduce BMI<25 kg/m^2^ and waist circumference <102cm in men, <88cm in women)

- Moderation of alcohol intake (men: no more than 2 units a day, women: 1 unit a day)

*Blood pressure and risk prediction chart*

The risk charts (see end of document) can be used to estimate the risk of developing a cardiovascular disease based on the blood pressure and other parameters. They should be used as guidance for starting medication according to the schedule below:

- Stage 1 Hypertension (>140/90mmHg - <160/100mmHg)

5 year CVD risk <10%: lifestyle and annually reassessed

5 year CVD risk 10-20%: after 3 months (including lifestyle change) without improvement > start medication

5 year CVD risk >20%: start medication if BP >130/80 mmHg

- Stage 2 Hypertension (>160/100mmHg)

Independent of risk: start medication

*Medication*

**Antihypertensive medication:**

First choice: Hydrochlorothiazide (25-50mg p.o once daily)

Second choice: Nifedipine (20-40mg p.o twice daily)

Third choice: Enalapril (5-20mg p.o. twice daily)

In combination with diabetes first choice is Enalapril.

Main side effects:

Hydrochlorothiazide: vertigo, muscle cramps, dry mouth, gout, increased sugar

Nifedipine: palpitations, oedema and headaches

Enalapril: dry cough, increase of potassium levels, swelling lips and tongue (stop direct)

Metformin: abdominal pain, nausea, loose bowel motions

Based on side-effects you might decide to change medication. Avoid using other medication without first consulting a medical doctor.

*Follow-up*

Follow up with medication should be every month in the first three to six months. When target blood pressure is reached (BP<140/90 or BP 130/80 for CVD risk>20%) appointments can be reduced to every three to six months.

It is advised that the patient should be accompanied to the clinic by a household member so that lifestyle adjustments and adherence to medication will be facilitated.

At every follow-up visit, progress with lifestyle changes should be discussed, side effects assessed and BMI + BP measured and Morisky Scale to measure adherence, see below.

*Referral:*

- Clinical features of a hypertensive emergency (high blood pressure plus restlessness, anxiety, agitation, headache, paralysis, blurred vision, vomiting, dyspnoea)

- Clinical features of heart disease e. g heart failure, angina, arrhythmia

- History of previous stroke/TIA

- Suspicion of secondary hypertension disease e.g. oedema, urine reduced or blood/protein

- Severe hypertension (BP >200/120) not managed on the same day

- Hypertension in pregnancy

- Severe diabetes not controlled with metformine

- Lack of control while on medication

- Once the condition of the above categories of people is assessed and stabilized, they can be followed up in a primary care facility based on the recommendations provided in these guidelines. They will however need periodic reassessments in specialty care.

**Morisky Scale:**

1. Do you ever forget to take your medicine?
2. Are you careless at times about taking your medicine?
3. When you feel better do you sometimes stop taking your medicine?
4. Sometimes if you feel worse when you take the medicine, do you stop taking it?

Interpretation Morisky Scale: score 1 point for every YES answer

0 points = high adherence

1-2 points = intermediate

3-4 points = low adherence
